# Evaluating a Land Use Regression Model for Estimating Metals in Fine Particulate Matter across the Denver Metro Area: The Healthy Start Study

**DOI:** 10.1021/acsestair.5c00325

**Published:** 2026-02-19

**Authors:** Anne Mielnik, Sheena E. Martenies, Christian L’Orange, Anne P. Starling, William B. Allshouse, John L. Adgate, Grace Kuiper, Sherry WeMott, Dana Dabelea, Sheryl Magzamen

**Affiliations:** † Department of Environmental and Radiological Health Sciences, 3447 Colorado State University, Fort Collins, Colorado 80523-1019, United States; ‡ Department of Health and Kinesiology, 14589 University of Illinois Urbana−Champaign, Urbana, Illinois 61801-3028, United States; § Department of Mechanical Engineering, Colorado State University, Fort Collins, Colorado 80523-1019, United States; ∥ Department of Epidemiology, 144805 Colorado School of Public Health, University of Colorado Anschutz Medical Campus, Aurora, Colorado 80045, United States; ⊥ Lifecourse Epidemiology of Adiposity and Diabetes (LEAD) Center, University of Colorado Anschutz Medical Campus, Aurora, Colorado 80045, United States; # Department of Epidemiology, 41474 University of North Carolina, Chapel Hill, North Carolina 27599-7400, United States; ¶ Department of Environmental and Occupational Health, Colorado School of Public Health, University of Colorado Anschutz Medical Campus, Aurora, Colorado 80045, United States; ∇ Department of Pediatrics, School of Medicine, University of Colorado Anschutz Medical Campus, Aurora, Colorado 80045, United States

**Keywords:** air pollution, particulate matter, metals, environmental modeling, land use regression

## Abstract

Few studies examine health effects of metals in ambient fine particulate matter (PM_2.5_), as measurements of elemental composition are sparse. To facilitate intraurban studies in Denver, Colorado, we developed land use regression models for seven species—copper (Cu), iron (Fe), titanium (Ti), zinc (Zn), potassium (K), calcium (Ca), and magnesium (Mg). As part of the Healthy Start Cohort study, we collected filter-based PM_2.5_ using personal air samplers at 67 locations across Denver. Sample collection occurred from May 2018 through March 2019, accounting for all meteorological seasons. Exposure models were informed by 83 geospatial covariates, with traffic-related predictors as the strongest and most consistent across models. Model performance was evaluated using 10-fold cross validation and overall, varied by sampling campaign and season, with *R* ^2^ values ranging from 0 to 0.63. At best, our model predicts Cu and Fe during fall (*R* ^2^ = 0.56 and 0.63, respectively); whereas it fails to capture species unrelated to traffic year-round (*R* ^2^ < 0.40). This highlights the influence of missing predictors (e.g., wildfire smoke, atmospheric transport and other meteorological factors) on PM_2.5_ concentrations and spatial gradients. Despite limitations, resulting models enable estimation of intraurban metal exposures and support future analyses of long-term health impacts in Denver.

## Graphical abstract

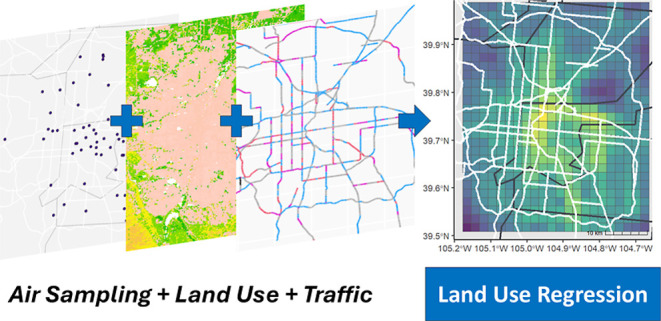

## Introduction

Airborne particulate matter (PM) is a complex mixture of solids and aerosols composed of metals, elemental and organic carbon, and other species.^1^ Particles with a diameter of 2.5 μm or less (PM_2.5_) are inhalable and deposit in the respiratory tract, posing risk to health.^1^ Short- and long-term PM_2.5_ exposures are associated with adverse health outcomes, including exacerbations of heart and lung conditions, neurological disorders, and premature death.^2–11^ PM_2.5_ originates from both anthropogenic (e.g., fossil fuel burning) and biogenic (e.g., woodburning) sources.^1^ Further, previous studies have identified nontailpipe vehicle emissions as an important source of metals in PM_2.5_, with copper (Cu) and barium (Ba) associated with brake wear and zinc (Zn) with tire wear.^12–14^ Metals in PM are toxic due to their potential to form reactive oxygen species, which are associated with biological mechanisms underlying disease, including oxidative stress, inflammation, immune responses, gene disruption, and cell death.^15,16^ While many studies have established a relationship between PM_2.5_ mass and health effects, fewer have examined the role of elemental composition on health, as measurements of PM_2.5_ elemental composition, including metals, are sparse, occurring sub weekly at 150 monitors nationwide.^6,17–19^ In Colorado, these data are collected at only one federal monitoring station.^17^ Thus, spatially refined estimates of PM_2.5_ metal concentrations are needed for intraurban health effect studies focused on local sources within the Denver metro area.

Land use regression (LUR) is a statistical modeling approach commonly used for environmental exposure assessment, including traffic-related air pollutants such as PM_2.5_ and nitrogen dioxide (NO_2_).^20–23^ Yet, few studies have been conducted to estimate exposure to individual PM_2.5_ species.^24–30^ In general, intraurban studies that examine PM_2.5_ species best capture nontailpipe vehicle emissions near roadways for all PM fractions, with traffic and railways as the strongest predictors.^24–28^ Most recently, Yin et al.^24^ leveraged data from Southern California to develop exposure models for coarse elemental (PM_10–2.5_), specific and total PM_2.5_, best capturing Cu, Fe, and Zn near roadways for all PM fractions (*R* ^2^ = 0.76 to 0.92). In the same region, Liu et al.^25^ also modeled nontailpipe vehicle emissions by cokriging and LUR using a low-cost sensor network, best estimating Ba (*R* ^2^ = 0.60) and, to a lesser extent, PM_2.5_ mass and Zn (*R* ^2^ = 0.41 and 0.47, respectively). Similarly, in Toronto, Canada, Cu and Fe concentrations were predominantly explained by traffic and railways (*R* ^2^ = 0.68 to 0.79) and estimated to be highest during summer.^26^ Interestingly, in New York, NY and Pittsburgh, PA, regional point source emissions (e.g., residual oil burning and steel-related emissions) were the strongest predictors of ambient PM_2.5_ species concentrations.^27,28^ LUR has also been used to evaluate modeling capabilities between different cities and/or study areas.^29,30^ Although previous studies find limited commonality in key predictors across models for different urban areas, land use, traffic, and vegetation are significant in predicting PM species across North America and Europe; with model performance for Cu, Fe, and Zn in PM_10–2.5_ ranging from *R* ^2^ = 0.36 to 0.86.^29,30^ Notably, exposure models evaluated in our study rely on low-cost measurements distributed throughout Denver to best represent local land use characteristics, enhancing the spatial coverage of PM_2.5_ species measurements compared to federal reference-grade monitors.^31^

A general limitation for exposure models used to estimate metals in air is the cost of sample collection and analysis for PM elemental composition. To elaborate, the current “gold standard” consists of collecting filter-based PM samples and analyzing elemental composition using Inductively Coupled Plasma Mass Spectrometry (ICP–MS). However, recurrent sample collection of filter-based measurements is time- and labor-intensive, and deployment of ICP–MS poses challenging instrument and labor demands. Further, ICP–MS is expensive, costing up to $500,000 USD per instrument, which may limit feasibility in field-based epidemiological studies. Alternatively, energy dispersive X-ray fluorescence (EDXRF) is also used to analyze elemental composition of PM_2.5_. EDXRF is well-established and routinely used in regulatory and research contexts for analysis of ambient PM_2.5_ elemental composition, including in the U.S. EPA Chemical Speciation Network (CSN) and IMPROVE network.^17,32^ Further, EPA Method IO-3.3 and IMPROVE protocols validate EDXRF for metal species most relevant to ambient PM exposure assessment.^32,33^ Compared to ICP–MS, EDXRF is (1) nondestructive, allowing for reanalysis of collected filter samples, (2) easier to use, requiring less training for personnel, and (3) less expensive, costing less than $100,000 USD per instrument. Therefore, to address these challenges, we used air pollution field campaign data from the Healthy Start cohort of the Environmental influences on Child Health Outcomes (ECHO) study to estimate metal concentrations in PM_2.5_ across Denver, CO. Briefly, ECHO is a prebirth cohort study that investigates impacts of prenatal and early life factors, including air pollution and other environmental exposures, on pediatric health outcomes.^34–36^ In this work, we developed and evaluated LUR models to predict concentrations of metal species in ambient PM_2.5_. Our models incorporate filter-based personal air sampling with EDXRF measurements from unique sites across Denver, CO and 83 geospatial covariates, including roadway information, traffic data, and land use. Predictions were generated for seven metal species, including Cu, Fe, titanium (Ti), Zn, potassium (K), calcium (Ca), and magnesium (Mg). Nontailpipe vehicle emissions (e.g., Cu, Fe, Ti, and Zn) are of particular interest, as they are unregulated and comprise a growing fraction of traffic-related PM.^37^

## Materials and Methods

### Particulate Matter Sampling and Elemental Composition Analysis

Over the course of four sampling campaigns (Campaign 1: May 8 to July 2, 2018; Campaign 2: July 10 to August 27, 2018; Campaign 3: October 9 to November 19, 2018; Campaign 4: January 22 to March 12, 2019), we collected filter-based PM_2.5_ using Ultrasonic Personal Air Samplers (UPAS; Access Sensor Technologies, Fort Collins, CO) at 67 locations across Denver, CO (Figure 1). Sample collection occurred over 5.5 day periods and was assumed representative of the season of deployment. PM_2.5_ mass was quantified gravimetrically (DS XS3DU Microbalance, Mettler-Toledo, Columbus, OH). EDXRF (ARL QUANT’X EDXRF Spectrometer, Thermo Scientific, Waltham, MA) was used for analysis of elemental composition. We calculated the limit of detection for PM_2.5_ mass to be three times the standard deviation of field blank measurements. Minimum detection limits (MDLs) were determined on an element-specific basis and based on instrument variability and photon counting statistics (Table S1). In XRF analysis, an element was considered detected when its photon emissions were distinguished from background noise with sufficient confidence. Thus, elements with measurements that were not at least 2 times above relative uncertainty were treated as below the MDL and were not used in statistical analysis.

**Figure 1:**
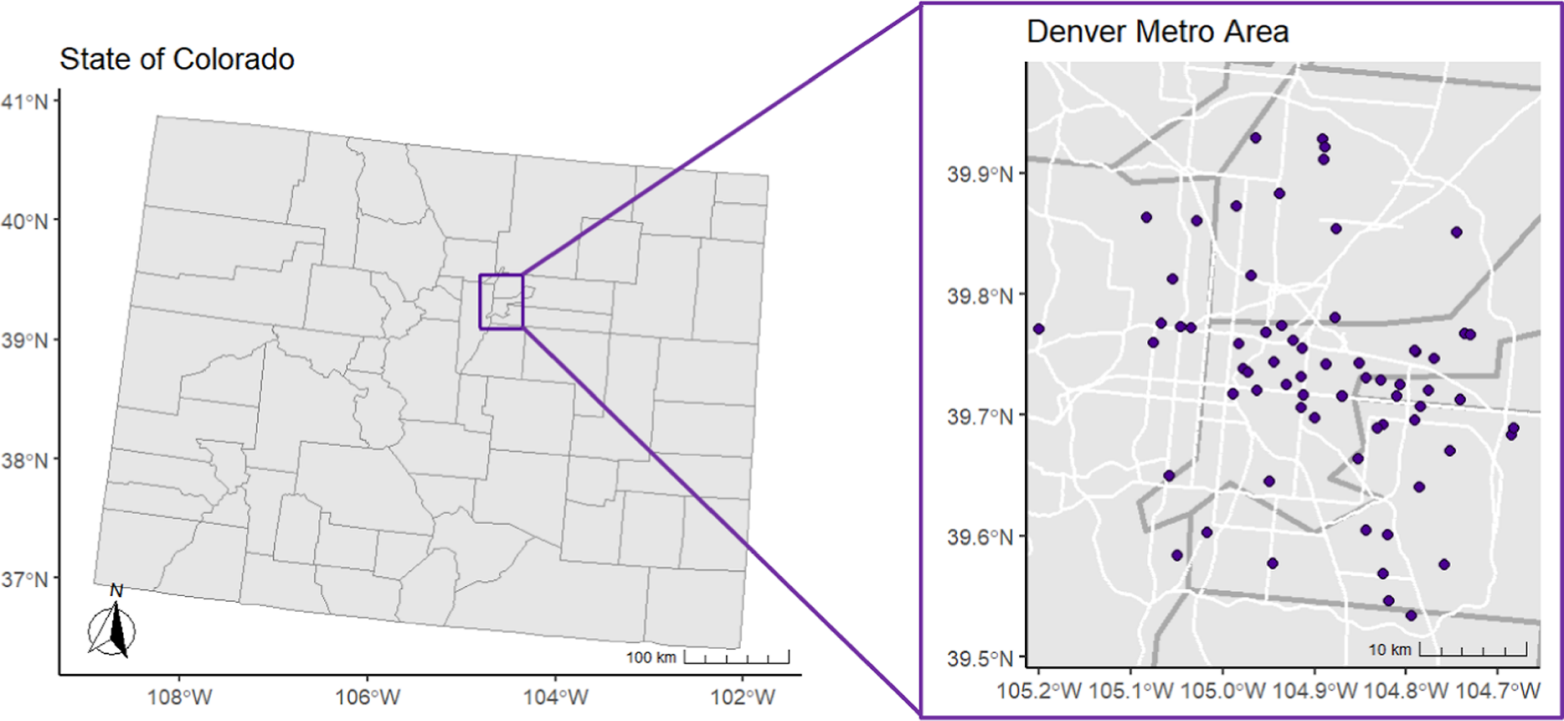
Map of Colorado with county lines (left) and the Denver Metro Area (right) with county lines (gray), highways (white), and filter-based personal air sampler measurements (purple). Sampling locations were jittered by 4.2 km to protect study participant privacy.

Concentrations of 24 PM_2.5_ species (e.g., Na, Mg, Al, Si, S, Cl, K, Ca, Ti, Cr, Fe, Ni, Cu, Zn, Ga, As, Se, Cd, In, Sn, Te, I, Pb, and Mn) were calculated using areal densities (in μg cm^–2^) detected by EDXRF, area of the polytetrafluoroethylene filter (MTL Corporation, Minneapolis, MN) equipped with UPAS (area = 7.065 cm^2^), and volume of sampled air. Detailed information about site selection, sample collection, and quantification of filter-based measurements is reported elsewhere.^31^ In this study, samples were excluded if (1) measurements fell below MDLs of EDXRF, (2) UPAS malfunctioned or provided incomplete information during the sampling period, (3) filters were located indoors, or (4) measured concentrations were outliers.

To elaborate, due to the large number of nondetects for most species, model predictions were generated by excluding values below MDLs. Replacing these measurements with zero or a calculated, fixed value (e.g., MDL/2 or MDL/√2) introduced bias into the data set, skewing the distribution toward zero and reducing variation. Overall, the inclusion of these measurements weakened predictor-outcome relationships and decreased R^2^ values for each model run. Thus, all nondetects were removed. Outliers were identified using the interquartile range (IQR) method, where observations outside of the following range for each species were considered outliers: Lower limit = Q1 – 1.5*IQR, upper limit = Q3 + 1.5*IQR. As a result, model predictions were generated for seven of 24 species detected: Cu, Fe, Ti, Zn, K, Ca, and Mg.

### Predictor Variables

Predictors are primarily composed of land use-related variables and are listed in Table S2. Predictor variables consist of estimates at exact locations within the study area (points) or of averages or sums of variables within designated radii around sampling sites (buffers). For each predictor evaluated as a buffer, multiple buffer variables were created, ranging from 50 to 2500 m in radius. Predictors in the data set include, but are not limited to elevation, impervious surfaces, land use, population count and density, tree cover, distance to nearest/emissions from stationary point sources, traffic, distance to nearest/length of highways and major roads, distance to nearest/length of railways, etc. Predictors were chosen a priori, based on previous LUR models for traffic-related air pollutants and PM.^31^ Point estimates and buffer variables were calculated using R v. 4.4.0.^38^

### Statistical Analysis

LUR models were developed using Least Absolute Shrinkage and Selection Operator (LASSO) variable selection (glmnet package in R v. 4.4.0), which has been used to model air pollution in Denver and elsewhere.^22,29,31,38–40^ For LASSO modeling, we used long-term mean PM_2.5_ species concentrations (i.e., measurements from the entire study period) at each sampling location as the outcome. Mean concentrations subset by sampling campaign and meteorological season (i.e., Spring: March, April, May; Summer: June, July, August; Fall: September, October, November; Winter: December, January, February) were also considered as outcomes. All data for PM_2.5_ species concentrations were log transformed prior to fitting models. LUR models were fit over a 2 km grid across the Denver metro area (∼1968 km^2^ in total area). Model performance was evaluated by calculating root mean squared error (RMSE), mean absolute error (MAE), and r-squared (*R* ^2^) values using 10-fold cross validation (CV), selecting the best LASSO model for each PM_2.5_ species. PM_2.5_ species lacking measurements in at least two of four sampling campaigns (i.e., falling below MDL more than 50% of the study period) were excluded from analysis. Pearson correlation coefficients were calculated between measured PM_2.5_ species and select predictor variables and between modeled PM_2.5_ species using base R v. 4.4.0.^38^

## Results and Discussion

### Elemental Composition of PM_2.5_

Throughout the sampling period, we used 810 of 1293 weekly measurements from all 67 sites across the Denver metro area. As reported in Martenies et al.,^31^ median sampling time per filter was 5 days, ranging from 2 to 6 days, and median (range) number of samples collected at each site was 11 (2 to 17), with the highest number collected during Campaign 4 (*n* = 366), followed by Campaign 1 (*n* = 341), Campaign 2 (*n* = 338), and Campaign 3 (*n* = 248). The distribution of measurements is shown in Figure S1. As expected, measurements of nontailpipe vehicle emissions (e.g., Cu, Fe, Ti, and Zn) were positively correlated with traffic-related variables (e.g., average annual daily traffic (AADT) and distance to nearest major road; ρ = 0.1 to 0.5), railway length (ρ = 0.1 to 0.4), and each other (ρ = 0.2 to 0.8); whereas Alkali and Alkaline Earth metals (e.g., K, Ca, and Mg) lacked a relationship with traffic (ρ = 0), but were directly related to each other (ρ = 1; Figure 2). In previous studies, measurements of PM_2.5_ species concentrations, specifically Cu, Fe, Ti, and Zn, were also associated with traffic.^12–14,24,25,28^ In some regions, measured concentrations of PM_2.5_ species had stronger correlations with regional emission sources.^24,28^ For instance, in Southern California, vanadium in PM_2.5_ was positively correlated with diesel and ship emissions, whereas sodium was associated with sea spray and crustal emissions in this region and elsewhere.^24,28^ In New York City, strong associations were found between residual oil burning and measured concentrations of nickel and Zn.^28^

**Figure 2:**
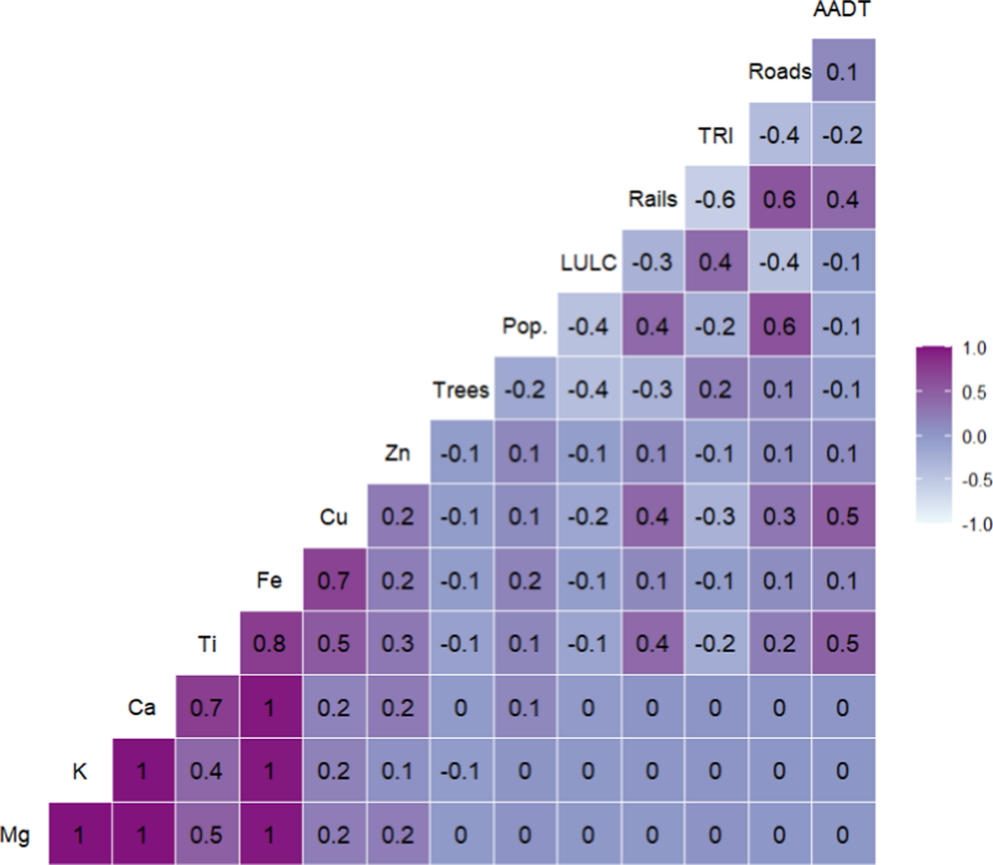
Pearson correlation coefficients among select predictor variables and measured PM_2.5_ species. Note: “Trees” refers to tree cover, “Pop.” is population density, “LULC” is land use, “Rails” is total railway length, “TRI” is total emissions from stationary point sources, “Roads” is total highway length, and “AADT” is average annual daily traffic. Select predictors shown here were calculated using buffer distance of 2500 m.

Notably, many samples fell below EDXRF MDLs (range: n = 0 nondetects for Ca, Mg, and Fe to n = 643 nondetects for As), limiting model predictions to seven of 24 species detected: Cu, Fe, Ti, Zn, K, Ca, and Mg. With this, measurements of most species fell beneath both instrument sensitivity and toxicologically meaningful exposure thresholds (e.g., 3 × 10^–5^ mg/m^3^ for Cr­(VI), 0.15 μg/m^3^ as a 3 month average for Pb); providing useful evidence that metals in ambient PM_2.5_ are unlikely to pose meaningful health concerns across Denver.^41,42^

### LUR Model Performance

Final LUR model performance and results from 10-fold CV are shown in Table 1. Predicted spatial distributions for the entire sampling period are shown in Figures 3 and 4 with estimations by campaign (Figures S2 and S3) and season (Figures S4 and S5). Predicted vs observed metal concentrations are shown in Figures 5 and 6. Overall, model performance varies by metal species, sampling campaign, and meteorological season. Significant predictor variables were inconsistent across models; however, for nontailpipe vehicle emissions (e.g., Cu, Fe, Ti, and Zn), the strongest predictors not only include traffic-related variables (e.g., AADT, heavy-duty traffic from buses and trucks, distance to nearest/length of highways and major roads, etc.), but also include distance to nearest/length of railways, and, to a lesser extent, distance to nearest/emissions from stationary point sources. Interestingly, land use was also significant in Ti models but was inversely related to ambient concentrations. This suggests developed areas (National Land Cover Database (NLCD) class 22–24) have the highest ambient Ti concentrations followed by forests (NLCD class 42), grasslands (NLCD class 71), agricultural areas (NLCD class 81–82), and then, wetlands (NLCD class 95). For Alkali and Alkaline Earth metals (e.g., Ca, Mg, and K) predictors varied more widely. Elevation and railway length were the strongest and most consistent predictors in Ca and Mg models; however, traffic-related variables played a seasonal role for both compounds, positively affecting Ca and Mg concentrations during fall and winter. Seasonal effects observed for Ca and Mg may be explained by practices such as road salting during winter months or by increased resuspension of dust during warmer, drier periods when vegetation cover is reduced and surface materials are more easily mobilized.^43,44^ Consistent predictors were not present in K models across sampling campaigns or seasons; however, this species is often associated with biomass burning, which is episodic and spatially variable, possibly explaining difficulty capturing K concentrations with the predictors in our model.^45,46^ At best, our model explains 63% of Fe variation in fall with RMSE of 0.667 ng/m^3^ and MAE of 0.406 ng/m^3^. Similarly, Cu variation is best captured in fall (*R* ^2^ = 0.56); whereas other nontailpipe vehicle emissions are best captured during wintertime or Campaign 4 (*R* ^2^ = 0.48 or 0.49 for Ti and *R* ^2^ = 0.47 for Zn). However, our model is less capable of capturing K, Ca, and Mg overall, by sampling campaign or season (*R* ^2^ < 0.40).

**Table 1:**
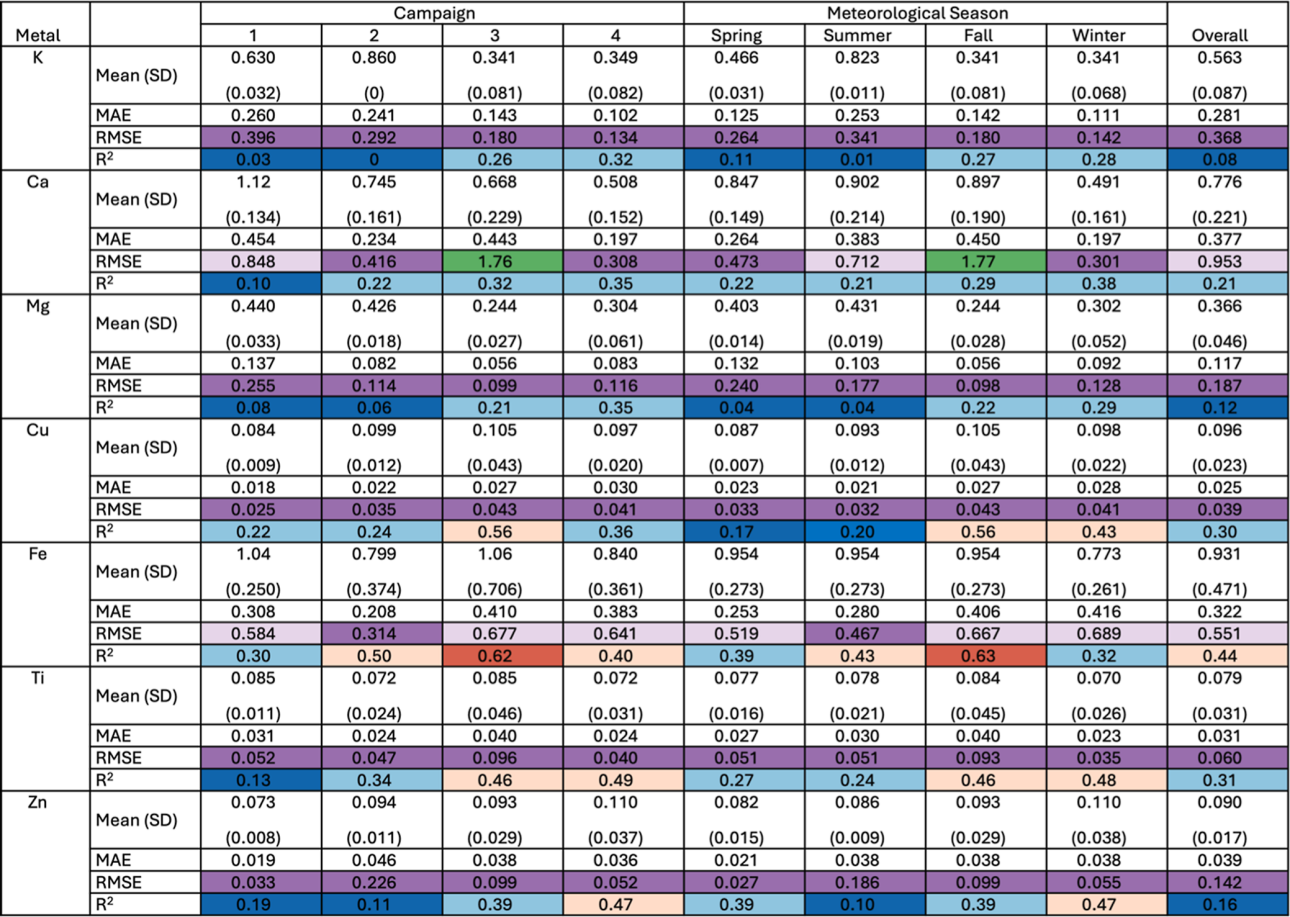
Summary Statistics and Model Performance for LUR Models for Select PM_2.5_ Species

**Figure 3:**
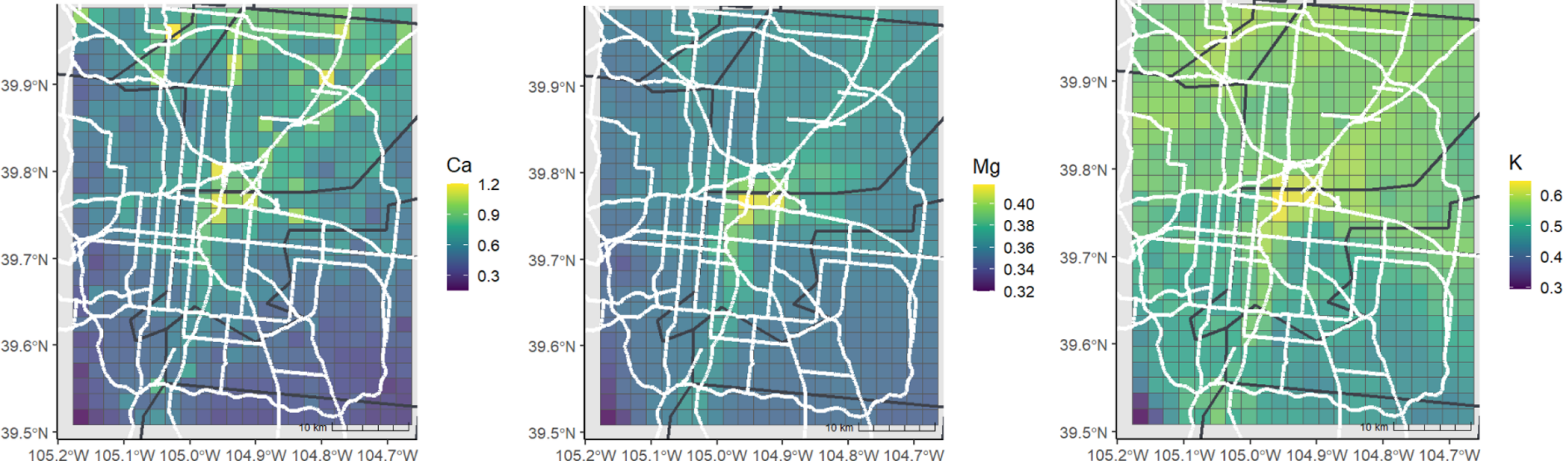
Estimated concentrations of Ca, Mg, and K for the entire study period, reported in units of ng/m^3^, shown with county lines (black) and highways (white).

**Figure 4:**
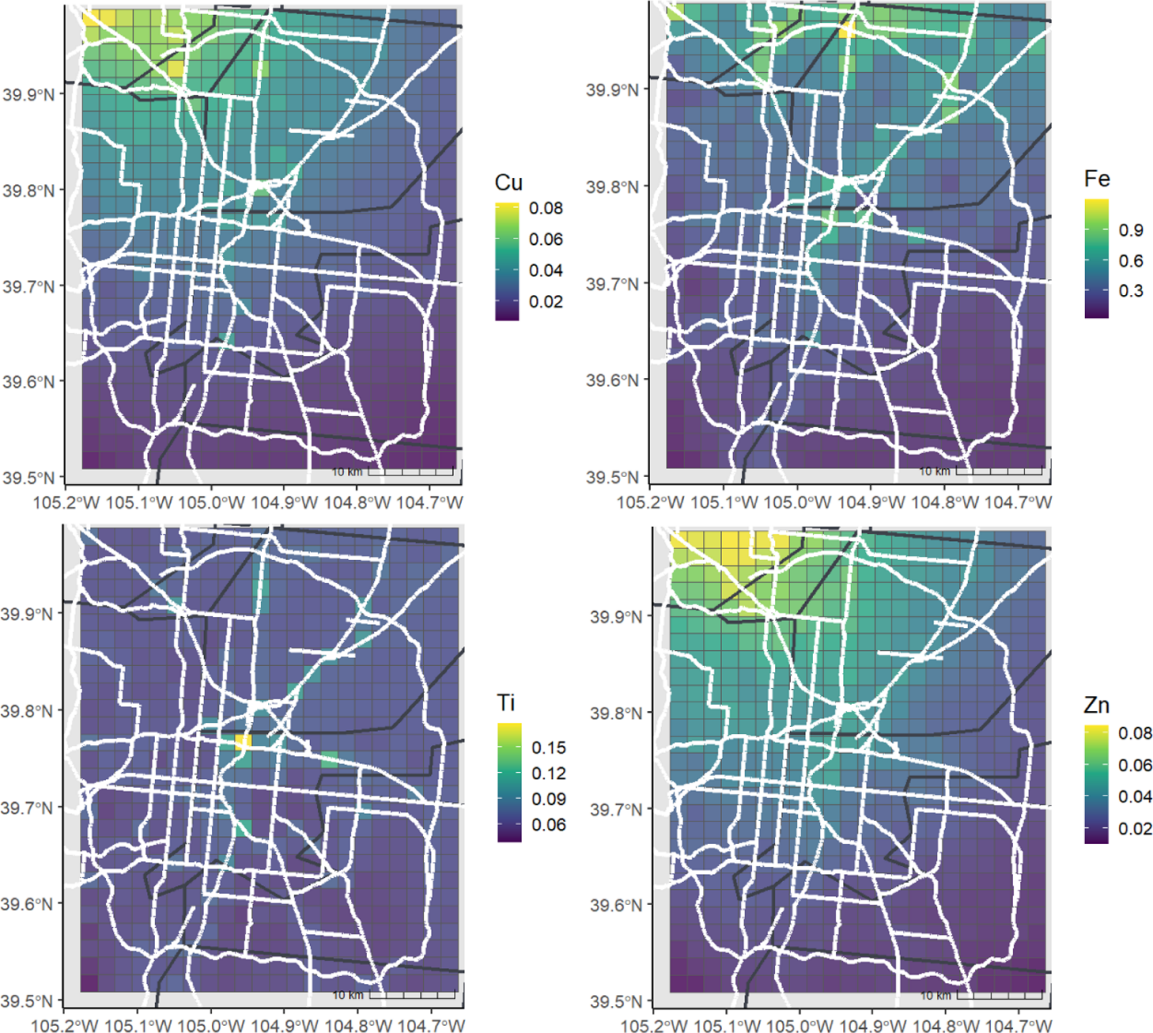
Estimated concentrations of Cu, Fe, Ti, and Zn for the entire study period, reported in units of ng/m^3^, shown with county lines (black) and highways (white).

**Figure 5:**
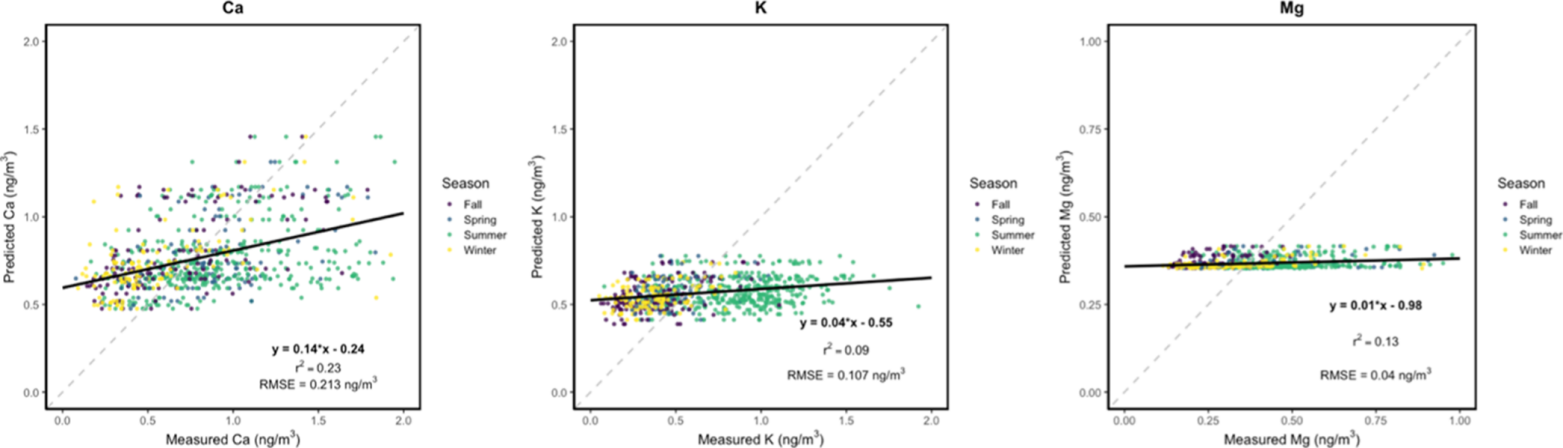
Measured vs predicted mean Ca, Mg, and K concentrations (in units of ng/m^3^), color-coded by meteorological season. *R* ^2^ values represent the square of correlations between direct Ca, Mg, and K measurements and respective model predictions for the entire sampling period. RMSE values are reported in ng/m^3^. Solid black lines represent regression lines and dashed gray lines represent 1:1 correspondence between measured and predicted concentrations.

**Figure 6:**
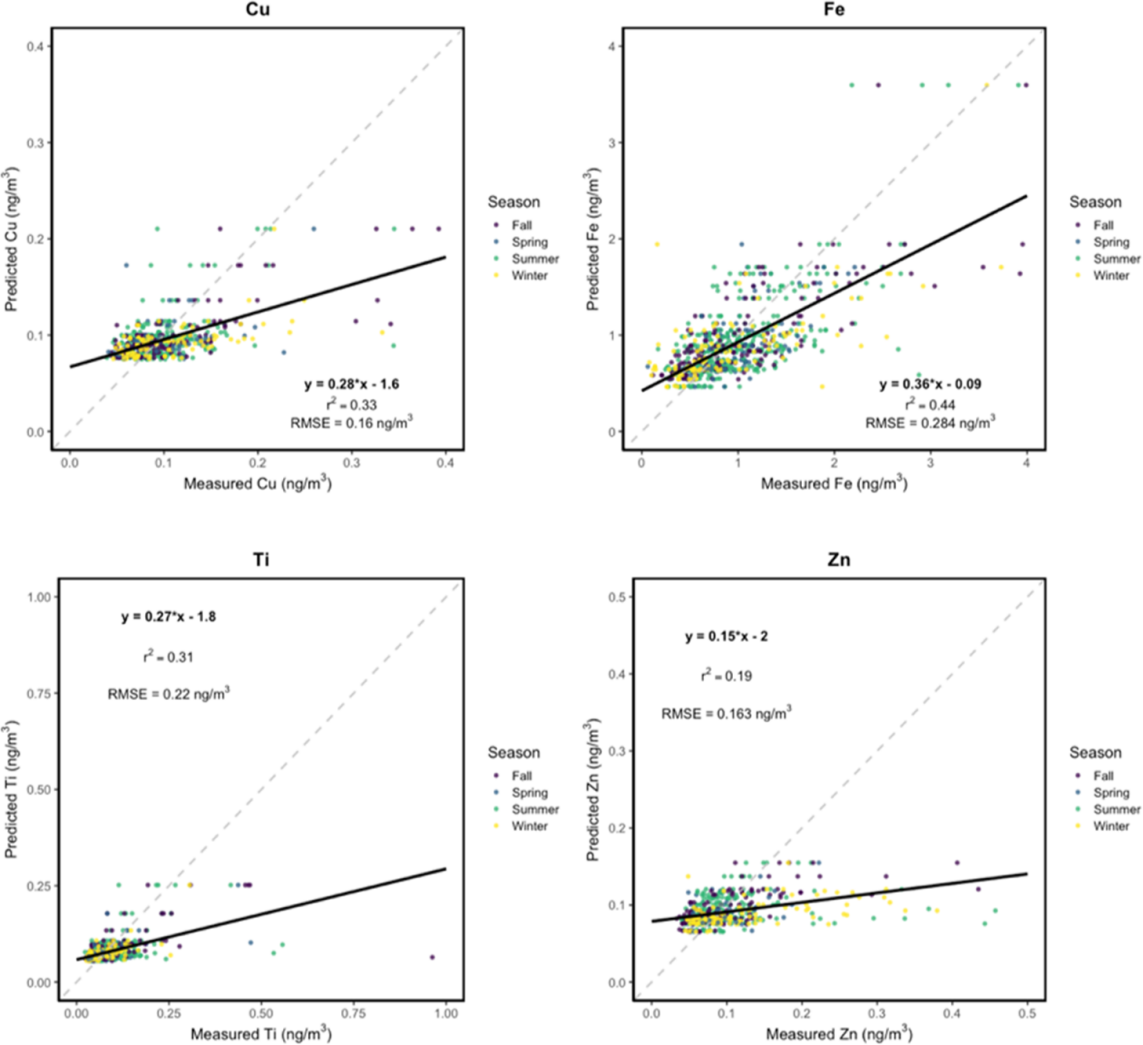
Measured vs predicted mean Cu, Fe, Ti, and Zn concentrations (in units of ng/m^3^), color-coded by meteorological season. *R* ^2^ values represent the square of correlations between direct Cu, Fe, Ti, and Zn measurements and respective model predictions for the entire sampling period. RMSE values are reported in ng/m^3^. Solid black lines represent regression lines and dashed gray lines represent 1:1 correspondence between measured and predicted concentrations.

“Campaign” refers to sampling campaign (Campaign 1: May 8 to July 2, 2018; Campaign 2: July 10 to August 27, 2018; Campaign 3: October 9 to November 19, 2018; Campaign 4: January 22 to March 12, 2019). Acronyms include mean absolute error (MAE), root mean squared error (RMSE), and standard deviation (SD). Mean, SD, MAE, and RMSE are reported in units of ng/m^3^. To represent low to high values, color scales range from dark blue (*R* ^2^ ≤ 0.2) to blue (0.2 < *R* ^2^ ≤ 0.4) to light orange (0.4 < *R* ^2^ ≤ 0.6) to orange (0.6 < *R* ^2^); and from purple (RMSE ≤0.5 ng/m^3^) to light purple (0.5 ng/m^3^ < RMSE ≤1 ng/m^3^) to green (1.5 ng/m^3^ < RMSE ≤2 ng/m^3^), with color groups missing for RMSE between 1 ng/m^3^ to 1.5 ng/m^3^ due to a lack of observations.

Although model performance is oftentimes modest (*r* ^2^ < 0.4) and thus, not ideal for health studies, it is comparable to other regional LUR models for PM_2.5_ species. For nontailpipe vehicle emissions, Fe, Cu, and Zn are the most studied.^24–30,39^ Whereas, only one prior publication has investigated Ti in PM_2.5_, to our knowledge.^28^ Overall, in previous studies, ambient Fe concentrations are modeled with *R* ^2^ ranging from 0.47 to 0.87 and RMSE of 0.23 ng/m^3^ to 500 ng/m^3^.^24,26–28,30^ In comparison, our LUR model performs well (*R* ^2^ = 0.30 to 0.63; RMSE = 0.314 ng/m^3^ to 0.689 ng/m^3^), accounting for less variance during certain seasons but providing more accuracy overall. In Southern California, Yin et al.^24^ found Fe in PM_2.5_ was associated with nontailpipe vehicle emissions, maintaining model performance with overall *R* ^2^ values of 0.73 and 0.79 (and RMSE of 0.35 and 0.37 ng/m^3^) using leave-one-out CV (LOOCV) and 10-fold CV, respectively. In New York, NY and Pittsburgh, PA, Fe was also most associated with traffic-related predictors and maintained adequate model performance using LUR (*R* ^2^ = 0.63 and 0.55, respectively).^27,28^ Similarly, in Toronto, Canada, Fe concentrations were predominantly explained by traffic and railways (*R* ^2^ = 0.79) and estimated to be highest during summer.^26^

Additionally, Cu has been modeled with *R* ^2^ values ranging from 0.47 to 0.86 (RMSE: 0.22 ng/m^3^ to 30 ng/m^3^).^24–27,30,39^ In our study, *R* ^2^ = 0.17 to 0.56 and RMSE = 0.025 ng/m^3^ to 0.043 ng/m^3^, which is comparable to previous regional LUR models. Specifically, Yin et al.^24^ also found Cu was associated with nontailpipe vehicle emissions and achieved excellent model performance (overall *R* ^2^ = 0.78 and 0.87 with RMSE = 0.34 and 0.29 ng/m^3^ using LOOCV and 10-fold CV, respectively). In New York, NY, Cu was also associated with traffic-related predictors and maintained model performance of *R* ^2^ = 0.58 to 0.68.^28^ Similarly, in Toronto, Canada, Cu concentrations were predominantly explained by traffic and railways (*R* ^2^ = 0.68) and estimated to be highest during summer.^26^

Interestingly, Zn has been modeled with *R* ^2^ values ranging from 0.36 to 0.80 (RMSE: 0.28 ng/m^3^ to 25 ng/m^3^).^24–27,30,39^ In our study, *R* ^2^ = 0.10 to 0.47 and RMSE = 0.027 ng/m^3^ to 0.226 ng/m^3^. In Southern California, Zn was associated with nontailpipe vehicle emissions, maintaining model performance with overall *R* ^2^ values of 0.66 and 0.76 (and RMSE of 0.53 and 0.57 ng/m^3^) using LOOCV and 10-fold CV, respectively.^24^ In the same region, Liu et al.^25^ found Zn was most associated with distance to railways and traffic-related predictors, maintaining *R* ^2^ of 0.47. In Pittsburgh, PA, Zn was best predicted by steel mill-specific emissions (model *R* ^2^ = 0.37) whereas in New York, NY, Zn was most associated with residual oil burning (model *R* ^2^ = 0.54 to 0.80).^27,28^ Overall, our exposure models capture less spatial variance in Fe, Cu, and Zn concentrations compared to other regional studies, yet demonstrate more accuracy in predicting Fe, Cu, and Zn concentrations, as shown by lower *R*_2_ and RMSE values, respectively.

Lastly, Ito et al.^28^ modeled Ti concentrations in New York City, reporting *R* ^2^ of 0.49, which is comparable to our findings for the Denver metro area (*R* ^2^ = 0.13 to 0.49). In general, our LUR models comparable to previous studies, but may be missing important predictors that account for spatial variation, specifically meteorological factors including wind speed and direction, atmospheric stability, temperature, relative humidity, and boundary layer height. These data were excluded because available measurements are not spatially representative of the study area, with only 12 monitoring sites across Denver.^47^ Also, our PM_2.5_ samples were collected over 5.5 day periods, whereas meteorological measurements are reported hourly and vary throughout the day—the effects of which we cannot capture with our measurements. Prior work with higher *R* ^2^ and lower RMSE values either (1) directly implemented meteorology data into exposure models or (2) used a hybrid LUR approach, incorporating estimations of metals or other air pollutant concentrations from dispersion models into LUR models.^24,25,27,29^ Thus, meteorology and atmospheric conditions (i.e., the physical factors underlying dispersion modeling) may be relevant pollutant-specific parameters that influence spatial variation in the Denver metro area.

Compared to nontailpipe vehicle emissions, Alkali and Alkaline Earth metals are less commonly studied, likely due to their lack of known health effects. Nonetheless, in previous studies, K models have been developed for New York City and 20 locations across Europe, capturing spatial variation well (*R* ^2^ = 0.64 and 0.45, respectively), unlike our K models (*R* ^2^ = 0 to 0.32).^28,30^ Also, to our knowledge, only one other study modeled Ca, but in PM_10–2.5_, with a range in model performance by community in Southern California (e.g., *R* ^2^ = 0.17 for Santa Barbara vs *R* ^2^ = 0.83 for Mira Loma).^24^ As our model domain was not as large, we noticed more profound temporal differences, with model performance varying by sampling campaign and season (*R* ^2^ = 0.10 to 0.38). Although we did not find prior research on Mg in PM_2.5_, we noted that RMSE values for Ca (RMSE = 0.301 ng/m^3^ to 1.77 ng/m^3^) and K (RMSE = 0.134 ng/m^3^ to 0.396 ng/m^3^) were comparable to or several orders of magnitude lower than values reported previously. Yin et al.^24^ reported RMSE ranging from 0.16 ng/m^3^ to 0.41 ng/m^3^ for Ca in PM_10–2.5_ in Southern California, whereas de Hoogh et al.^30^ computed RMSE of 120 ng/m^3^ for K in PM_2.5_ across Europe. However, increased RMSE observed in the latter is likely reflective of the number of study areas included in the analysis.

### Elemental Correlations

We calculated pairwise Pearson correlation coefficients for modeled PM_2.5_ species (Figure 7). Correlations ranged from 0.4–1, indicating it may be possible to evaluate some species independently of others in health studies; however, for those that are highly correlated (ρ ≥ 0.7), health effects may be difficult to distinguish.

**Figure 7:**
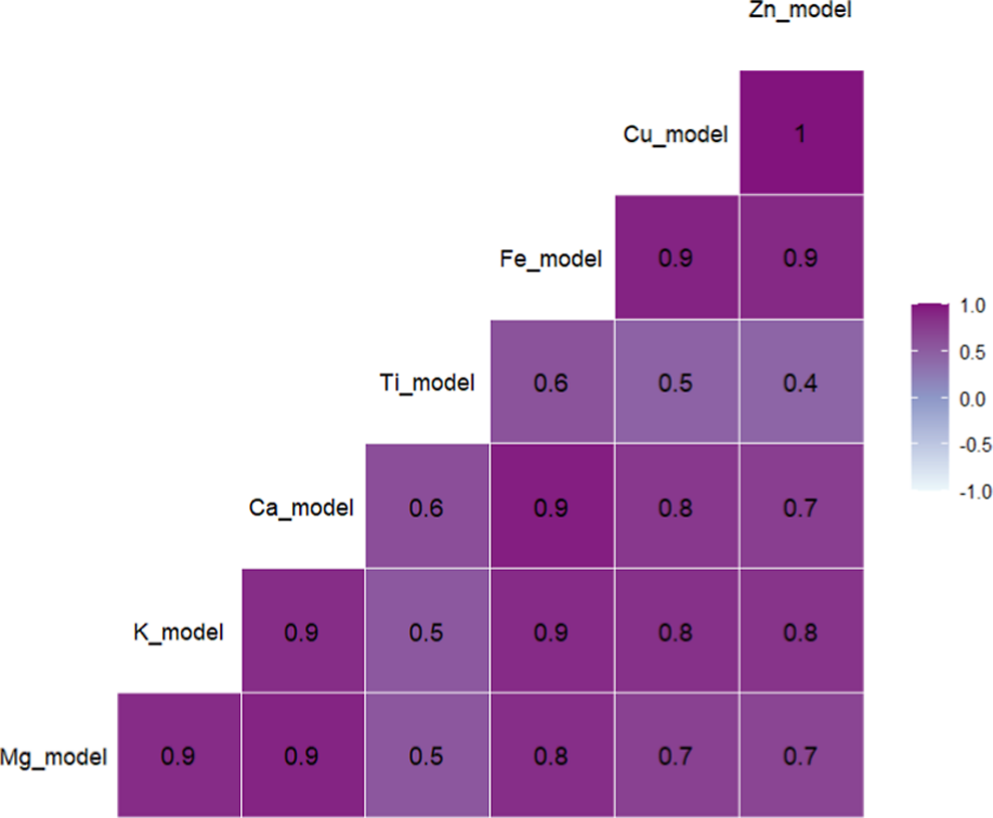
Pearson correlation coefficients among modeled PM_2.5_ species.

The relationship between heavy metals Cu, Fe, and Zn is notable (ρ ≥ 0.9) yet unsurprising, as these compounds are thought to come from nontailpipe vehicle emissions, specifically tire and brake wear particles.^12–14^ Similarly, Alkaline Earth metals Ca and Mg are highly correlated (ρ = 0.9), while Alkali metal K is also highly correlated to both these species (ρ = 0.9). Interestingly, heavy metal Ti is correlated to Fe (ρ = 0.6) yet less correlated to Cu and Zn (ρ = 0.5 and 0.4, respectively), potentially reflecting increased Fe and Ti concentrations from brake wear particles versus tire wear particles or traffic volume, as seen previously, or suggesting both compounds have another source in Denver.^12^

### Key Strengths and Limitations

The successful implementation of LUR modeling for metals in PM_2.5_ across the Denver metro area highlights key strengths of our approach. First, our models focus on PM_2.5_ elemental composition, including heavy metals, some of which are toxic and may have important health implications for the Healthy Start cohort and other populations in Denver. Second, the deployment of filter-based measurements allowed for repeated sampling across the study area, capturing spatial and temporal gradients necessary for health studies. Additionally, our sampling approach was relatively low-cost and enabled the determination of metals using EDXRF, capturing 24 different species in PM_2.5_. Third, LUR allowed us to estimate PM_2.5_ species concentrations at previously unmeasured locations, allowing intraurban comparisons. Lastly, to our knowledge, this is the first exposure assessment study to model PM_2.5_ species at residential locations in Denver, CO, a sparsely monitored region relative to other densely populated urban areas.

Despite these strengths, several limitations must be acknowledged. First, our study used EDXRF to analyze elemental composition instead of the “gold standard,” ICP–MS. While EDXRF is generally faster, less expensive, and nondestructive, ICP–MS offers higher selectivity and sensitivity for metal species and has lower detection limits than EDXRF. However, sampling via ICP–MS is more time- and labor-intensive compared to EDXRF, which directly translates to increased costs and potentially limits the number of samples that can be collected and analyzed.

Second, sample collection during this study occurred on a weekly basis for one year only, which did not provide enough longitudinal measurements of elemental composition for statistical models with higher time resolution (e.g., spatiotemporal modeling). Thus, we were only able to predict PM_2.5_ species concentrations and spatial gradients for the entire study period, by sampling campaign and season. This model would therefore not be appropriate for the investigation of short-term health outcomes (e.g., asthma exacerbations) or episodic events (e.g., wildfire smoke exposure). We could, however, employ this framework to examine long-term health outcomes, such as mortality, as done previously.^48^ However, these data were not collected during the Healthy Start Cohort study, as the study population was a prebirth cohort. In addition, previous studies that employ the LUR framework typically achieve better model performance.^21^ For example, Eeftens et al.^21^ developed LUR models for PM in 20 European cities, achieving median *R* ^2^ of 0.71 for PM_2.5_ (range 0.35 to 0.94). Final models were used to estimate particulate air pollutant concentrations at home addresses of participants in consequential health studies, ultimately revealing PM_2.5_ is associated with lung cancer.^49^ Although LUR has been successfully used in health studies with varying degrees of accuracy and predictive power, low *R* ^2^ values for exposure models may impact effect estimates for exposure-outcome relationships by biasing effect estimates toward the null, underestimating the association between exposure and outcome, and reducing overall statistical power—limiting the implementation of exposure models presented here. Thus, we recommend future models incorporate predictors that require higher time-resolution measurements (e.g., hourly or daily) such as wildfire smoke, atmospheric transport and meteorology, which are not captured here. Previous LUR models have shown increased *R* ^2^ values with the inclusion of these factors.^20,24^ Specifically, Yin et al.^24^ demonstrated the importance of including spatiotemporally resolved meteorology data in LUR models to improve performance for health studies. To our knowledge, it is the only study that includes both direct measurements of PM_2.5_ elemental composition and daily meteorology data and achieves the best model performance of all regional LUR models discussed here.

Further, we used the LUR framework to model PM_2.5_ elemental composition rather than newer, more robust modeling techniques (e.g., spatiotemporal modeling or machine learning), which have demonstrated better predictive power than LUR for PM_2.5_ composition.^31,50^ Previous work from the Healthy Start Cohort study successfully implemented spatiotemporal modeling to estimate black carbon (BC) exposure across Denver using similar data.^31^ However, this research maintained a critical assumption/strength that we could not leverage here. To elaborate, temporal trend functions needed to fit spatiotemporal models are derived using long-term monitoring data sets, typically from federal reference monitors. However, for both BC and PM_2.5_ elemental composition, only one regulatory monitor is active in Denver. To overcome this limitation, Martenies et al.^31^ relied on a priori knowledge and the strong correlation between BC and NO_2_ measurements (ρ = 0.7) to justify using 6 NO_2_ regulatory monitor measurements to fit temporal trend functions for BC spatiotemporal models in Denver. We did not find similar associations between PM_2.5_ elemental composition and NO_2_, and thus could not make the same assumption. Therefore, spatiotemporal modeling was not used for this study. Additionally, LUR was chosen over machine learning because: (1) although the measurements presented here were taken weekly at 67 unique locations, data were inconsistent across sampling sites, with median (range) number of samples collected at each site equal to 11 (2 to 17) over 44 weeks. We felt this lack of measurement data paired with a lack of temporally varying predictors would have been insufficient input for machine learning models, potentially resulting in underfitting, a lack of generalizability to our study area, and ultimately, an inability to learn new patterns (beyond relationships with traffic-related predictors) in the future.

Additionally, although subsetting EDXRF data by sampling campaign and meteorological season improved model performance in most instances, some exposure models suffered from overdispersion and as a result, overestimated PM_2.5_ species concentrations in certain locations. Finally, our use of LASSO for variable selection could potentially introduce positive bias into our CV measures, as this technique struggles with multicollinearity and, if variables are highly correlated, may arbitrarily drop predictors.

Despite these limitations, we successfully fit intraurban LUR models to estimate metal concentrations in PM_2.5_ across the Denver metro area. With this, we have developed the framework to assess long-term average PM_2.5_ species exposures and evaluate their associations with health outcomes in the Healthy Start cohort and other populations. Key takeaways of our study include, first, models developed here can be used to reliably estimate Cu, Fe, Ti, and Zn in PM_2.5_, which are heavy metals and have known adverse health effects, yet have not been modeled previously in this region.^6,15^ Second, air samples were collected at 67 locations across the Denver metro area, best capturing land use characteristics and enhancing spatial coverage of PM_2.5_ species measurements compared to existing monitoring data. Lastly, although traffic-related predictor variables were significant in all models, performance varied by season. This highlights the potential influence of omitted predictor variables (e.g., wildfire smoke, atmospheric transport and other meteorological factors) on PM_2.5_ concentrations and spatial gradients in this region.

## Supplementary Material



## Data Availability

Code used to calculate point estimates and buffers for predictor variables, create LUR models and evaluate model performance is available on the primary author’s GitHub page. Pollutant measurement data is available upon request.

## Notes

Additional information about EDXRF MDLs, geospatial predictor variables used in LUR models, the distribution of PM_2.5_ species measurements taken during the study period, and model output from exposure models by sampling campaign and meteorological season can be found here (PDF)

## References

[1] Inhalable Particulate Matter and Health (PM2.5 and PM10). https://ww2.arb.ca.gov/resources/inhalable-particulate-matter-and-health?keywords=2025 (accessed 04 28, 2025).

[2] Hayes R. B., Lim C., Zhang Y., Cromar K., Shao Y., Reynolds H. R., Silverman D. T., Jones R. R., Park Y., Jerrett M., Ahn J., Thurston G. D. (2020). PM2.5 Air Pollution and Cause-Specific Cardiovascular
Disease Mortality. Int. J. Epidemiol..

[3] Fan J., Li S., Fan C., Bai Z., Yang K. (2016). The Impact of PM2.5
on Asthma Emergency Department Visits: A Systematic Review and Meta-Analysis. Environ. Sci. Pollut. Res..

[4] Zhu R.-X., Nie X.-H., Chen Y.-H., Chen J., Wu S.-W., Zhao L.-H. (2020). Relationship Between Particulate
Matter (PM2.5) and
Hospitalizations and Mortality of Chronic Obstructive Pulmonary Disease
Patients: A Meta-Analysis. Am. J. Med. Sci..

[5] Han F., Yang X., Xu D., Wang Q., Xu D. (2019). Association
between Outdoor PM2.5 and Prevalence of COPD: A Systematic Review
and Meta-Analysis. Postgrad. Med. J..

[6] Badaloni C., Cesaroni G., Cerza F., Davoli M., Brunekreef B., Forastiere F. (2017). Effects of
Long-Term Exposure to Particulate Matter
and Metal Components on Mortality in the Rome Longitudinal Study. Environ. Int..

[7] Fu P., Guo X., Cheung F. M. H., Yung K. K. L. (2019). The Association between PM2.5 Exposure
and Neurological Disorders: A Systematic Review and Meta-Analysis. Sci. Total Environ..

[8] Shi L., Wu X., Danesh Yazdi M., Braun D., Abu Awad Y., Wei Y., Liu P., Di Q., Wang Y., Schwartz J., Dominici F., Kioumourtzoglou M.-A., Zanobetti A. (2020). Long-Term Effects of PM2·5 on
Neurological Disorders in the American Medicare Population: A Longitudinal
Cohort Study. Lancet Planet. Health.

[9] Peng W., Liu T. (2025). Greenness Modified
the Association of PM2.5 and Ozone with Global
Disease Burden of Alzheimer’s Disease and Other Dementias. Sci. Rep..

[10] Apte J. S., Marshall J. D., Cohen A. J., Brauer M. (2015). Addressing Global Mortality
from Ambient PM_2.5_. Environ. Sci.
Technol..

[11] Shiraiwa M., Ueda K., Pozzer A., Lammel G., Kampf C. J., Fushimi A., Enami S., Arangio A. M., Fröhlich-Nowoisky J., Fujitani Y., Furuyama A., Lakey P. S. J., Lelieveld J., Lucas K., Morino Y., Pöschl U., Takahama S., Takami A., Tong H., Weber B., Yoshino A., Sato K. (2017). Aerosol Health Effects from Molecular
to Global Scales. Environ. Sci. Technol..

[12] Wang J. M., Jeong C.-H., Hilker N., Healy R. M., Sofowote U., Debosz J., Su Y., Munoz A., Evans G. J. (2021). Quantifying
Metal Emissions from Vehicular Traffic Using Real World Emission Factors. Environ. Pollut..

[13] Oroumiyeh F., Jerrett M., Del Rosario I., Lipsitt J., Liu J., Paulson S. E., Ritz B., Schauer J. J., Shafer M. M., Shen J., Weichenthal S., Banerjee S., Zhu Y. (2022). Elemental
Composition of Fine and Coarse Particles across the Greater Los Angeles
Area: Spatial Variation and Contributing Sources. Environ. Pollut..

[14] Lopez B., Wang X., Chen L.-W. A., Ma T., Mendez-Jimenez D., Cobb L. C., Frederickson C., Fang T., Hwang B., Shiraiwa M., Park M., Park K., Yao Q., Yoon S., Jung H. (2023). Metal Contents
and Size Distributions
of Brake and Tire Wear Particles Dispersed in the Near-Road Environment. Sci. Total Environ..

[15] Valko M., Morris H., Cronin M. (2005). Metals, Toxicity
and Oxidative Stress. Curr. Med. Chem..

[16] Lodovici M., Bigagli E. (2011). Oxidative Stress and
Air Pollution Exposure. J. Toxicol..

[17] Chemical Speciation Network (CSN). https://www.epa.gov/amtic/chemical-speciation-network-csn (accessed 2025 04 02).

[18] Bell M. L., Dominici F., Ebisu K., Zeger S. L., Samet J. M. (2007). Spatial
and Temporal Variation in PM_2.5_ Chemical Composition in
the United States for Health Effects Studies. Environ. Health Perspect..

[19] Harrison R. M., Yin J. (2000). Particulate Matter in the Atmosphere: Which Particle Properties Are
Important for Its Effects on Health?. Sci. Total
Environ..

[20] Ma X., Zou B., Deng J., Gao J., Longley I., Xiao S., Guo B., Wu Y., Xu T., Xu X., Yang X., Wang X., Tan Z., Wang Y., Morawska L., Salmond J. (2024). A Comprehensive Review
of the Development of Land Use
Regression Approaches for Modeling Spatiotemporal Variations of Ambient
Air Pollution: A Perspective from 2011 to 2023. Environ. Int..

[21] Eeftens M., Beelen R., De Hoogh K., Bellander T., Cesaroni G., Cirach M., Declercq C., Dėdelė A., Dons E., De Nazelle A., Dimakopoulou K., Eriksen K., Falq G., Fischer P., Galassi C., Gražulevičienė R., Heinrich J., Hoffmann B., Jerrett M., Keidel D., Korek M., Lanki T., Lindley S., Madsen C., Mölter A., Nádor G., Nieuwenhuijsen M., Nonnemacher M., Pedeli X., Raaschou-Nielsen O., Patelarou E., Quass U., Ranzi A., Schindler C., Stempfelet M., Stephanou E., Sugiri D., Tsai M.-Y., Yli-Tuomi T., Varró M. J., Vienneau D., Klot S. V., Wolf K., Brunekreef B., Hoek G. (2012). Development of Land
Use Regression Models for PM_2.5_, PM_2.5_ Absorbance,
PM_10_ and PM_coarse_ in 20 European Study Areas;
Results of the ESCAPE Project. Environ. Sci.
Technol..

[22] Larkin A., Geddes J. A., Martin R. V., Xiao Q., Liu Y., Marshall J. D., Brauer M., Hystad P. (2017). Global Land Use Regression
Model for Nitrogen Dioxide Air Pollution. Environ.
Sci. Technol..

[23] Mielnik A., Martenies S. E., Heiderscheidt J. M., Su J., Ross Z., Jerrett M., Balmes J. R., Magzamen S. (2022). Location-Weighted Traffic-Related
Air Pollution and Asthma Symptoms in Urban Adolescents. Air Qual., Atmos. Health.

[24] Yin X., Franklin M., Fallah-Shorshani M., Shafer M., McConnell R., Fruin S. (2022). Exposure Models for Particulate Matter Elemental Concentrations in
Southern California. Environ. Int..

[25] Liu J., Banerjee S., Oroumiyeh F., Shen J., Del Rosario I., Lipsitt J., Paulson S., Ritz B., Su J., Weichenthal S., Lakey P., Shiraiwa M., Zhu Y., Jerrett M. (2022). Co-Kriging with a Low-Cost Sensor Network to Estimate
Spatial Variation of Brake and Tire-Wear Metals and Oxidative Stress
Potential in Southern California. Environ. Int..

[26] Weichenthal S., Shekarrizfard M., Kulka R., Lakey P. S. J., Al-Rijleh K., Anowar S., Shiraiwa M., Hatzopoulou M. (2018). Spatial Variations
in the Estimated Production of Reactive Oxygen Species in the Epithelial
Lung Lining Fluid by Iron and Copper in Fine Particulate Air Pollution. Environ. Epidemiol..

[27] Tripathy S., Tunno B. J., Michanowicz D. R., Kinnee E., Shmool J. L. C., Gillooly S., Clougherty J. E. (2019). Hybrid
Land Use Regression Modeling
for Estimating Spatio-Temporal Exposures to PM2.5, BC, and Metal Components
across a Metropolitan Area of Complex Terrain and Industrial Sources. Sci. Total Environ..

[28] Ito K., Johnson S., Kheirbek I., Clougherty J., Pezeshki G., Ross Z., Eisl H., Matte T. D. (2016). Intraurban
Variation of Fine Particle Elemental Concentrations in New York City. Environ. Sci. Technol..

[29] Zhang K., Larson T. V., Gassett A., Szpiro A. A., Daviglus M., Burke G. L., Kaufman J. D., Adar S. D. (2014). Characterizing Spatial
Patterns of Airborne Coarse Particulate (PM_10–2.5_) Mass and Chemical Components in Three Cities: The Multi-Ethnic
Study of Atherosclerosis. Environ. Health Perspect..

[30] De
Hoogh K., Wang M., Adam M., Badaloni C., Beelen R., Birk M., Cesaroni G., Cirach M., Declercq C., Dėdelė A., Dons E., De Nazelle A., Eeftens M., Eriksen K., Eriksson C., Fischer P., Gražulevičienė R., Gryparis A., Hoffmann B., Jerrett M., Katsouyanni K., Iakovides M., Lanki T., Lindley S., Madsen C., Mölter A., Mosler G., Nádor G., Nieuwenhuijsen M., Pershagen G., Peters A., Phuleria H., Probst-Hensch N., Raaschou-Nielsen O., Quass U., Ranzi A., Stephanou E., Sugiri D., Schwarze P., Tsai M.-Y., Yli-Tuomi T., Varró M. J., Vienneau D., Weinmayr G., Brunekreef B., Hoek G. (2013). Development of Land Use Regression
Models for Particle Composition in Twenty Study Areas in Europe. Environ. Sci. Technol..

[31] Martenies S. E., Keller J. P., WeMott S., Kuiper G., Ross Z., Allshouse W. B., Adgate J. L., Starling A. P., Dabelea D., Magzamen S. (2021). A Spatiotemporal Prediction Model for Black Carbon
in the Denver Metropolitan Area, 2009–2020. Environ. Sci. Technol..

[32] IMPROVE. https://airquality.ucdavis.edu/improve (accessed 2025 11 05).

[33] Method IO-3.3. https://www.epa.gov/sites/default/files/2019-11/documents/mthd-3-3.pdf (accessed 2025 11 05).

[34] Harrod C.
S., Chasan-Taber L., Reynolds R. M., Fingerlin T. E., Glueck D. H., Brinton J. T., Dabelea D. (2014). Physical Activity in
Pregnancy and Neonatal Body Composition: The Healthy Start Study. Obstet. Gynecol..

[35] Martenies S. E., Oloo A., Magzamen S., Ji N., Khalili R., Kaur S., Xu Y., Yang T., Bastain T. M., Breton C. V., Farzan S. F., Habre R., Dabelea D. (2024). Independent
and Joint Effects of Neighborhood-Level Environmental and Socioeconomic
Exposures on Body Mass Index in Early Childhood: The Environmental
Influences on Child Health Outcomes (ECHO) Cohort. Environ. Res..

[36] Buckley J. P., Barrett E. S., Beamer P. I., Bennett D. H., Bloom M. S., Fennell T. R., Fry R. C., Funk W. E., Hamra G. B., Hecht S. S., Kannan K., Iyer R., Karagas M. R., Lyall K., Parsons P. J., Pellizzari E. D., Signes-Pastor A. J., Starling A. P., Wang A., Watkins D. J., Zhang M., Woodruff T. J. (2020). Opportunities for
Evaluating Chemical
Exposures and Child Health in the United States: The Environmental
Influences on Child Health Outcomes (ECHO) Program. J. Exposure Sci. Environ. Epidemiol..

[37] Fussell J. C., Franklin M., Green D. C., Gustafsson M., Harrison R. M., Hicks W., Kelly F. J., Kishta F., Miller M. R., Mudway I. S., Oroumiyeh F., Selley L., Wang M., Zhu Y. (2022). A Review of Road Traffic-Derived
Non-Exhaust Particles: Emissions, Physicochemical Characteristics,
Health Risks, and Mitigation Measures. Environ.
Sci. Technol..

[38] R Core Team. R: A Language and Environment for Statistical Computing, 2024. https://www.R-project.org/(accessed 2025 04 02).

[39] Zhang J. J.
Y., Sun L., Barrett O., Bertazzon S., Underwood F. E., Johnson M. (2015). Development of Land-Use Regression
Models for Metals Associated with Airborne Particulate Matter in a
North American City. Atmos. Environ..

[40] Friedman J., Hastie T., Tibshirani R. (2010). Regularization
Paths for Generalized
Linear Models via Coordinate Descent. J. Stat.
Softw..

[41] IRIS Toxicological Review of Hexavalent Chromium [Cr(VI)]. https://iris.epa.gov/static/pdfs/0144_summary.pdf. 39869745

[42] National Ambient Air Quality Standards (NAAQS) for Lead (Pb). https://www.epa.gov/lead-air-pollution/national-ambient-air-quality-standards-naaqs-lead-pb#rule-summary (accessed 2025 11 05).

[43] Aldrin M., Hobæk Haff I., Rosland P. (2008). The Effect of Salting with Magnesium
Chloride on the Concentration of Particular Matter in a Road Tunnel. Atmos. Environ..

[44] Litschke T., Kuttler W. (2008). On the Reduction of
Urban Particle Concentration by
Vegetation a Review. Z. Meteorol..

[45] Li J., Pósfai M., Hobbs P. V., Buseck P. R. (2003). Individual Aerosol
Particles from Biomass Burning in Southern Africa: 2, Compositions
and Aging of Inorganic Particles. J. Geophys.
Res.:Atmos..

[46] Pio C. A., Legrand M., Alves C. A., Oliveira T., Afonso J., Caseiro A., Puxbaum H., Sanchez-Ochoa A., Gelencsér A. (2008). Chemical Composition of Atmospheric
Aerosols during
the 2003 Summer Intense Forest Fire Period. Atmos. Environ..

[47] Pre-Generated Data Files. https://aqs.epa.gov/aqsweb/airdata/download_files.html (accessed 2025 06 04).

[48] Hart J. E., Garshick E., Dockery D. W., Smith T. J., Ryan L., Laden F. (2011). Long-Term Ambient Multipollutant
Exposures and Mortality. Am. J. Respir. Crit.
Care Med..

[49] Raaschou-Nielsen O., Andersen Z. J., Beelen R., Samoli E., Stafoggia M., Weinmayr G., Hoffmann B., Fischer P., Nieuwenhuijsen M. J., Brunekreef B., Xun W. W., Katsouyanni K., Dimakopoulou K., Sommar J., Forsberg B., Modig L., Oudin A., Oftedal B., Schwarze P. E., Nafstad P., De Faire U., Pedersen N. L., Östenson C.-G., Fratiglioni L., Penell J., Korek M., Pershagen G., Eriksen K. T., Sørensen M., Tjønneland A., Ellermann T., Eeftens M., Peeters P. H., Meliefste K., Wang M., Bueno-de-Mesquita B., Key T. J., De Hoogh K., Concin H., Nagel G., Vilier A., Grioni S., Krogh V., Tsai M.-Y., Ricceri F., Sacerdote C., Galassi C., Migliore E., Ranzi A., Cesaroni G., Badaloni C., Forastiere F., Tamayo I., Amiano P., Dorronsoro M., Trichopoulou A., Bamia C., Vineis P., Hoek G. (2013). Air Pollution and Lung Cancer Incidence in 17 European Cohorts: Prospective
Analyses from the European Study of Cohorts for Air Pollution Effects
(ESCAPE). Lancet Oncol..

[50] Lee Y. S., Choi E., Park M., Jo H., Park M., Nam E., Kim D. G., Yi S.-M., Kim J. Y. (2023). Feature Extraction
and Prediction of Fine Particulate Matter (PM2.5) Chemical Constituents
Using Four Machine Learning Models. Expert Syst.
Appl..

