# Supplementary material for: Evaluating a Land Use Regression Model for Estimating Metals in Fine Particulate Matter across the Denver Metro Area: The Healthy Start Study
Source: ACS EST Air. 2026 Feb 19;3(3):670–80. doi: 10.1021/acsestair.5c00325 (PMC12993808; doi:10.1021/acsestair.5c00325)
Supplement: Supplementary file 1 [file ea5c00325_si_001.pdf]

Supporting Information for

Evaluating a land use regression model for estimating metals in fine particulate matter across the Denver metro area: The Healthy Start Study

*Anne Mielnik,[1] Sheena E. Martenies,[1,2] Christian L'Orange,[3] Anne P. Starling,[4,5,6] William B. Allshouse,[7] John L. Adgate,[7] Grace Kuiper,[1] Sherry WeMott,[1] Dana Dabelea[4,5,8] & Sheryl Magzamen[1,4,5]**

1. Department of Environmental and Radiological Health Sciences, Colorado State University, Fort Collins, Colorado 80523-1019, United States
2. Department of Health and Kinesiology, University of Illinois Urbana-Champaign, Urbana, Illinois 61801-3028, United States
3. Department of Mechanical Engineering, Colorado State University, Fort Collins, Colorado 80523-1019, United States
4. Department of Epidemiology, Colorado School of Public Health, University of Colorado Anschutz Medical Campus, Aurora, Colorado 80045, United States
5. Lifecourse Epidemiology of Adiposity and Diabetes (LEAD) Center, University of Colorado Anschutz Medical Campus, Aurora, Colorado 80045, United States
6. Department of Epidemiology, University of North Carolina, Chapel Hill, North Carolina 27599-7400, United States
7. Department of Environmental and Occupational Health, Colorado School of Public Health, University of Colorado Anschutz Medical Campus, Aurora, Colorado 80045, United States
8. Department of Pediatrics, School of Medicine, University of Colorado Anschutz Medical Campus, Aurora, Colorado 80045, United States

*Corresponding Author:

Sheryl Magzamen
Department of Environmental and Radiological Health Sciences
Colorado State University
1618 Campus Delivery
Fort Collins, CO 80523-1618
sheryl.magzamen@colostate.edu

Supporting information (9 pages) includes 2 supplemental tables and 5 supplemental figures.

Table S1. Minimum detection limit (MDL) values for elements detected by EDXRF.

Element	MDL (µg/cm^2)
Al	0.006
As	0.006
Ca	0.004
Cd	0.093
Cl	0.006
Cr	0.005
Cu	0.008
Fe	0.004
Ga	0.005
I	0.315
In	0.109
K	0.002
Mg	0.005
Mn	0.013
Na	0.012
Ni	0.004
Pb	0.032
S	0.003
Se	0.018
Si	0.007
Sn	0.184
Te	0.217
Ti	0.009
Zn	0.002

MDL = 3 x C x √(B) / P where C is the concentration of standard used, B is background counts, and P is peak counts.

Table S2. Summary of spatial predictor variables considered for LUR models.

Variable	Units	Source	Type	Buffers (m)
Elevation	Mean within buffer (m)	NLCD	Raster	50, 100, 250, 500, 1000, 2500
Impervious surfaces	Mean within buffer (%)	NLCD	Raster	50, 100, 250, 500, 1000, 2500
Land use	Mode within buffer	NLCD	Raster	50, 100, 250, 500, 1000, 2500
Population count	Mean within buffer	NASA SEDAC	Raster	50, 100, 250, 500, 1000, 2500
Population density	Mean within buffer (Number of persons per km^2)	NASA SEDAC	Raster	50, 100, 250, 500, 1000, 2500
Tree cover	Mean within buffer (%)	NLCD	Raster	50, 100, 250, 500, 1000, 2500
Stationary point source emissions	Distance to nearest point source (m)	EPA TRI	Vector (Point)	
	Total emissions within buffer (lbs. per yr.)	EPA TRI	Vector (Point)	50, 100, 250, 500, 1000, 2500
Airports	Distance to nearest (m)	COIT	Vector (Point)	
CAFOs	Distance to nearest (m)	COIT	Vector (Point)	
Composting facilities	Distance to nearest (m)	COIT	Vector (Point)	
Landfills	Distance to nearest (m)	COIT	Vector (Point)	
Military installations	Distance to nearest (m)	COIT	Vector (Point)	
Mines	Distance to nearest (m)	COIT	Vector (Point)	
Oil and gas wells	Distance to nearest (m)	COIT	Vector (Point)	
Parks	Distance to nearest (m)	COIT	Vector (Point)	
WWTPs	Distance to nearest (m)	COIT	Vector (Point)	
AADT (Highways)	Mean AADT	CDOT/ HPMS	Vector (Linestring)	50, 100, 250, 500, 1000, 2500
AADT Single (Highways)	Mean AADT for single-unit trucks and buses (vehicle classes 4-7)	CDOT/ HPMS	Vector (Linestring)	50, 100, 250, 500, 1000, 2500
AADT Combined (Highways)	Mean AADT for combination trucks (vehicle classes 8-13)	CDOT/ HPMS	Vector (Linestring)	50, 100, 250, 500, 1000, 2500

Variable	Units	Source	Type	Buffers (m)
Highways	Distance to nearest (m)	CDOT	Vector (Linestring)	
	Total length within buffer (m)	CDOT	Vector (Linestring)	50, 100, 250, 500, 1000, 2500
Local roads	Distance to nearest (m)	CDOT	Vector (Linestring)	
Major roads	Distance to nearest (m)	CDOT	Vector (Linestring)	
	Total length within buffer (m)	CDOT	Vector (Linestring)	50, 100, 250, 500, 1000, 2500
Railways	Distance to nearest (m)	COIT	Vector (Linestring)	
	Total length within buffer (m)	COIT	Vector (Linestring)	50, 100, 250, 500, 1000, 2500

Acronyms: Confined animal feeding operation (CAFO), wastewater treatment plant (WWTP), average annual daily traffic (AADT), National Land Cover Dataset (NLCD), National Aeronautics and Space Administration Socioeconomic Data and Applications Center (NASA SEDAC), Environmental Protection Agency Toxic Release Inventory (EPA TRI), Colorado Governor's Office of Information Technology (COIT), Colorado Department of Transportation (CDOT), Federal Highway Administration Highway Performance Monitoring System (HPMS).

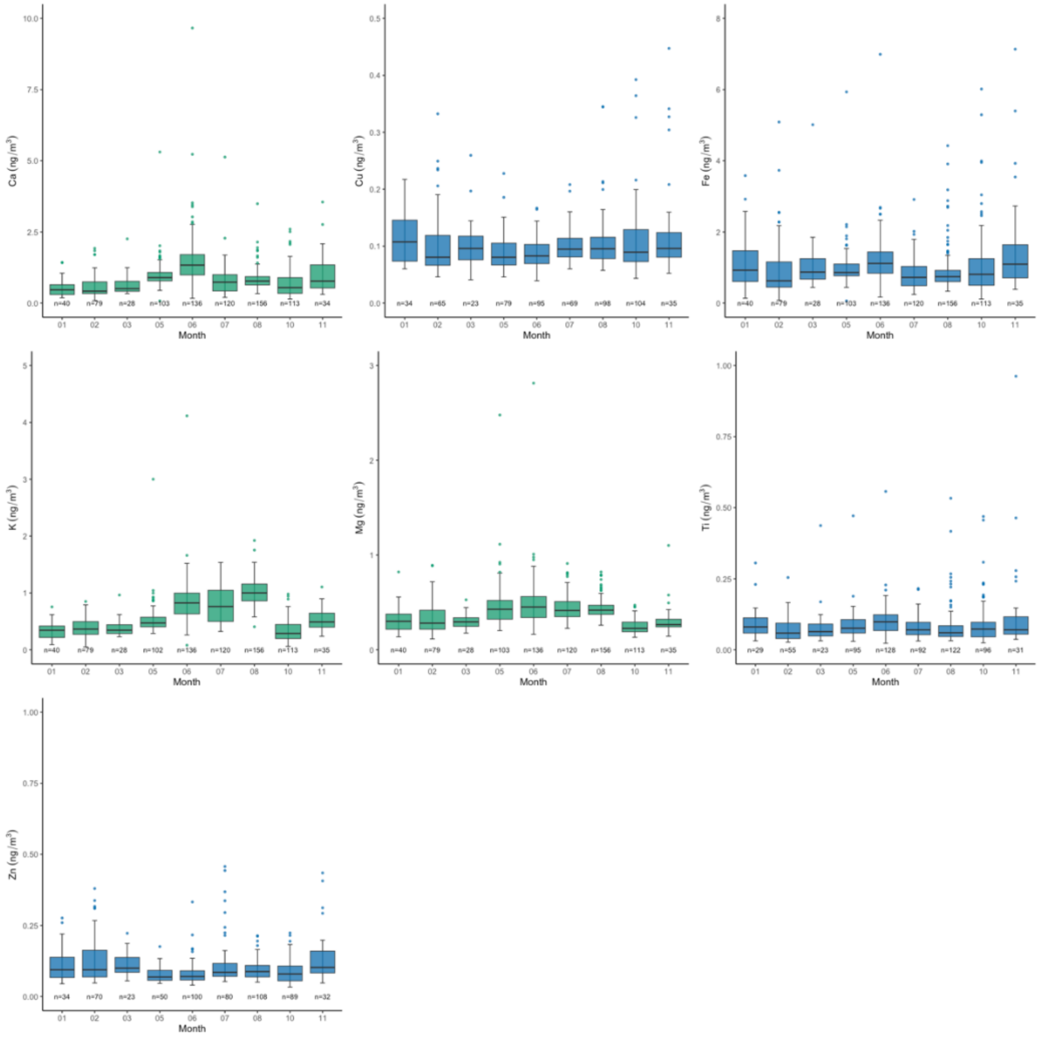

Figure S1. Distribution of measured $PM_{2.5}$ species concentrations, reported in units of ng/m^3. Months are labeled numerically (e.g., "01" is January, "06" is June, etc.) Heavy metals are shown in blue whereas Ca, Mg, and K are shown in green. Note: Y-axis scales were modified to highlight temporal trends, excluding outliers.

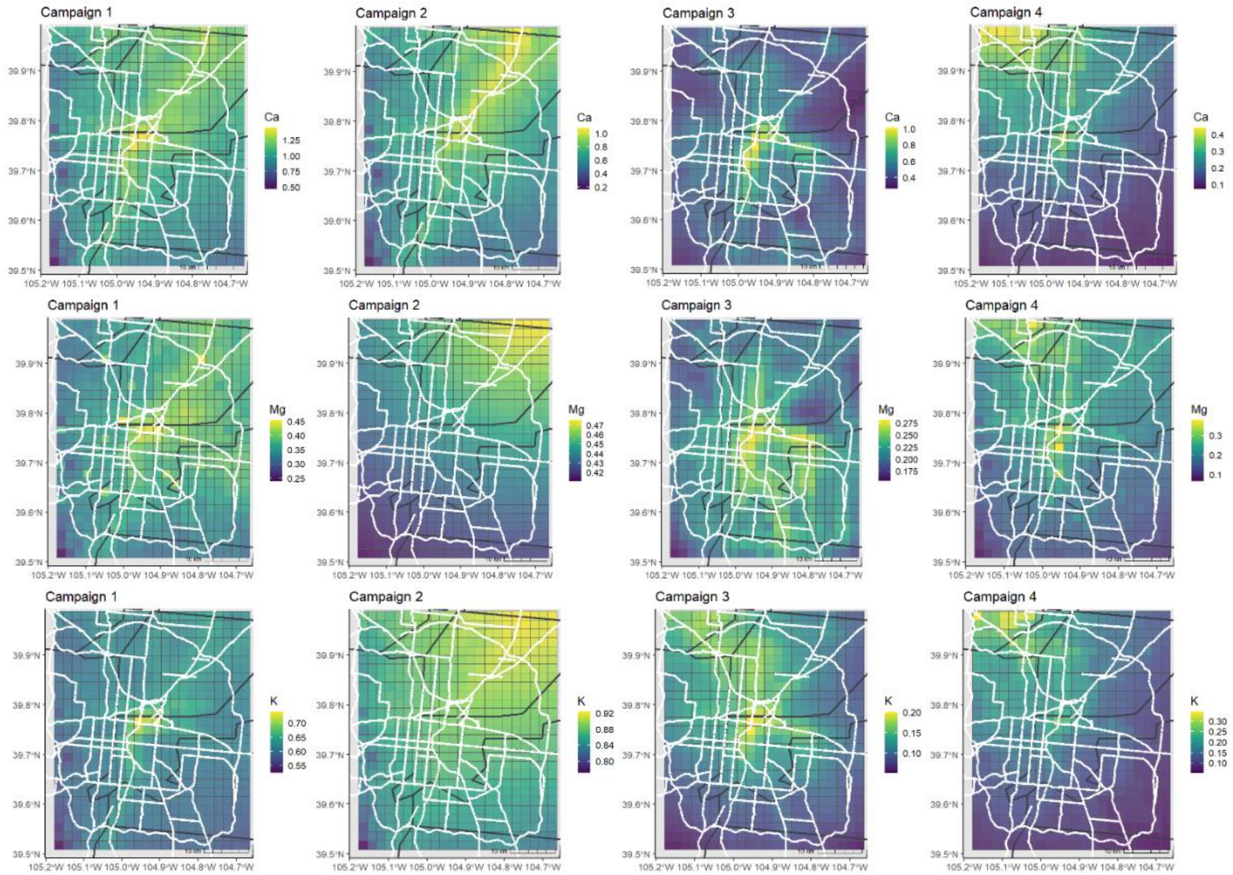

Figure S2. Estimated concentrations of Ca, Mg, and K by sampling campaign, reported in units of ng/m^3, shown with county lines (black) and highways (white).

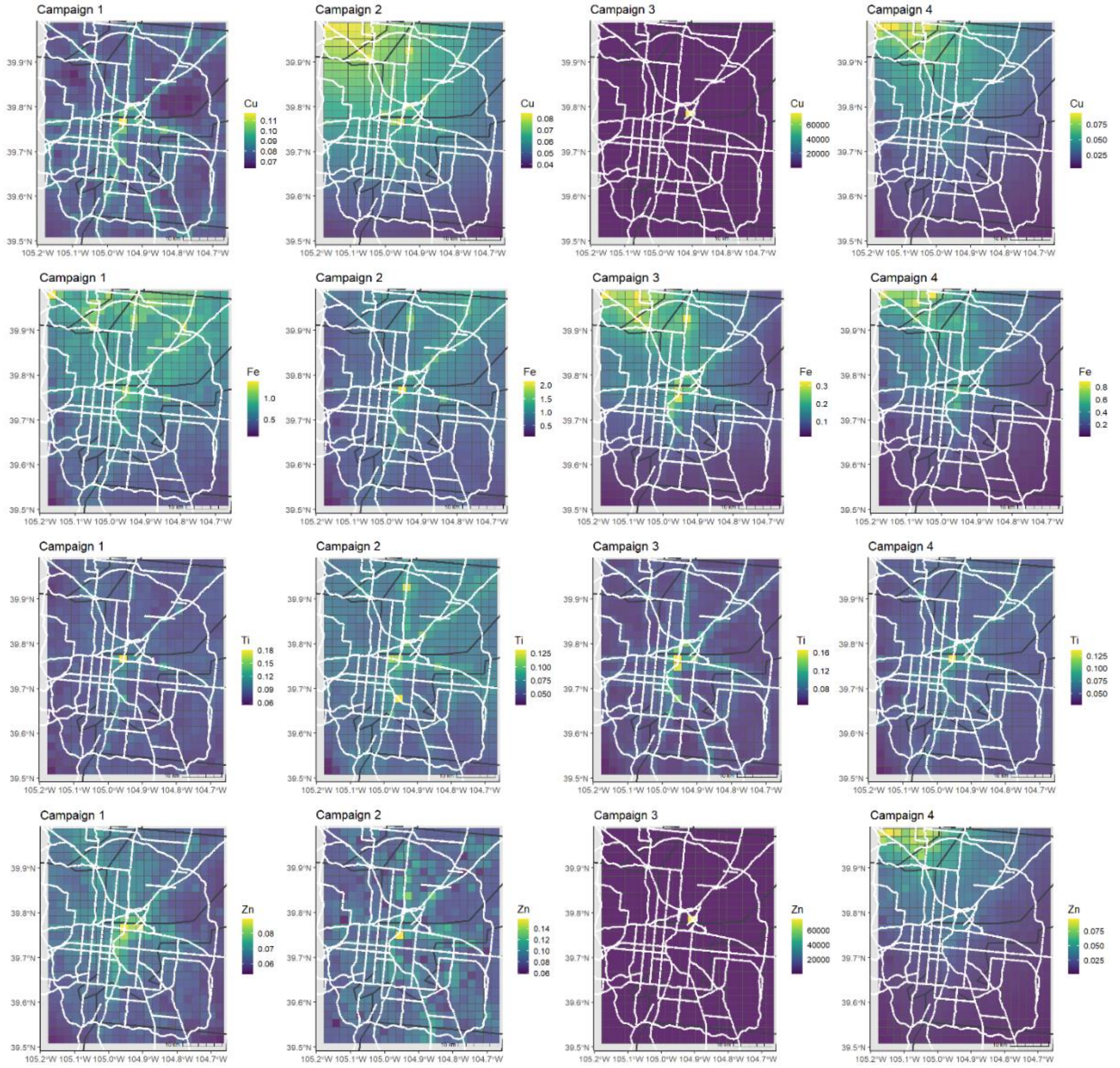

Figure S3. Estimated concentrations of Cu, Fe, Ti, and Zn by sampling campaign, reported in units of ng/m^3, shown with county lines (black) and highways (white).

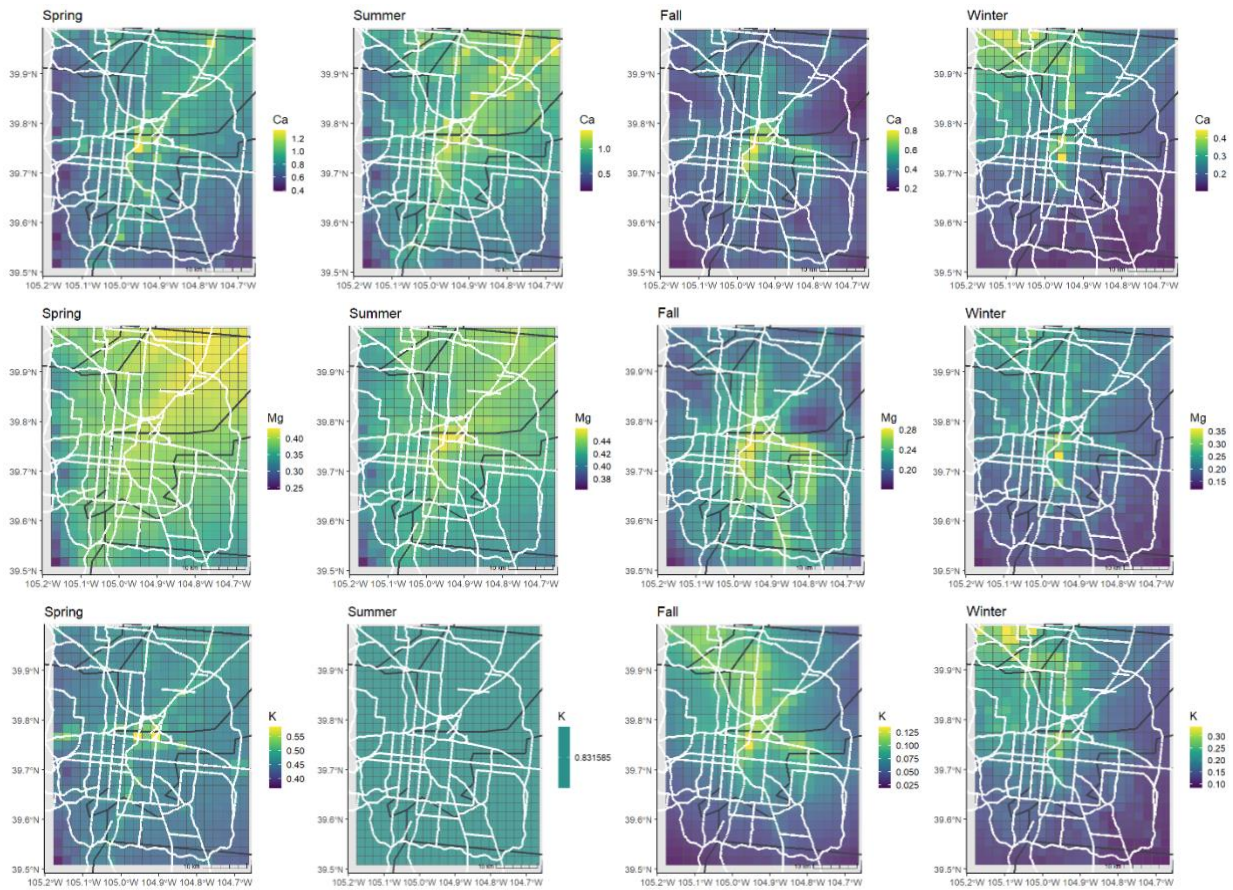

Figure S4. Estimated concentrations of Ca, Mg, and K by meteorological season, reported in units of ng/m^3, shown with county lines (black) and highways (white).

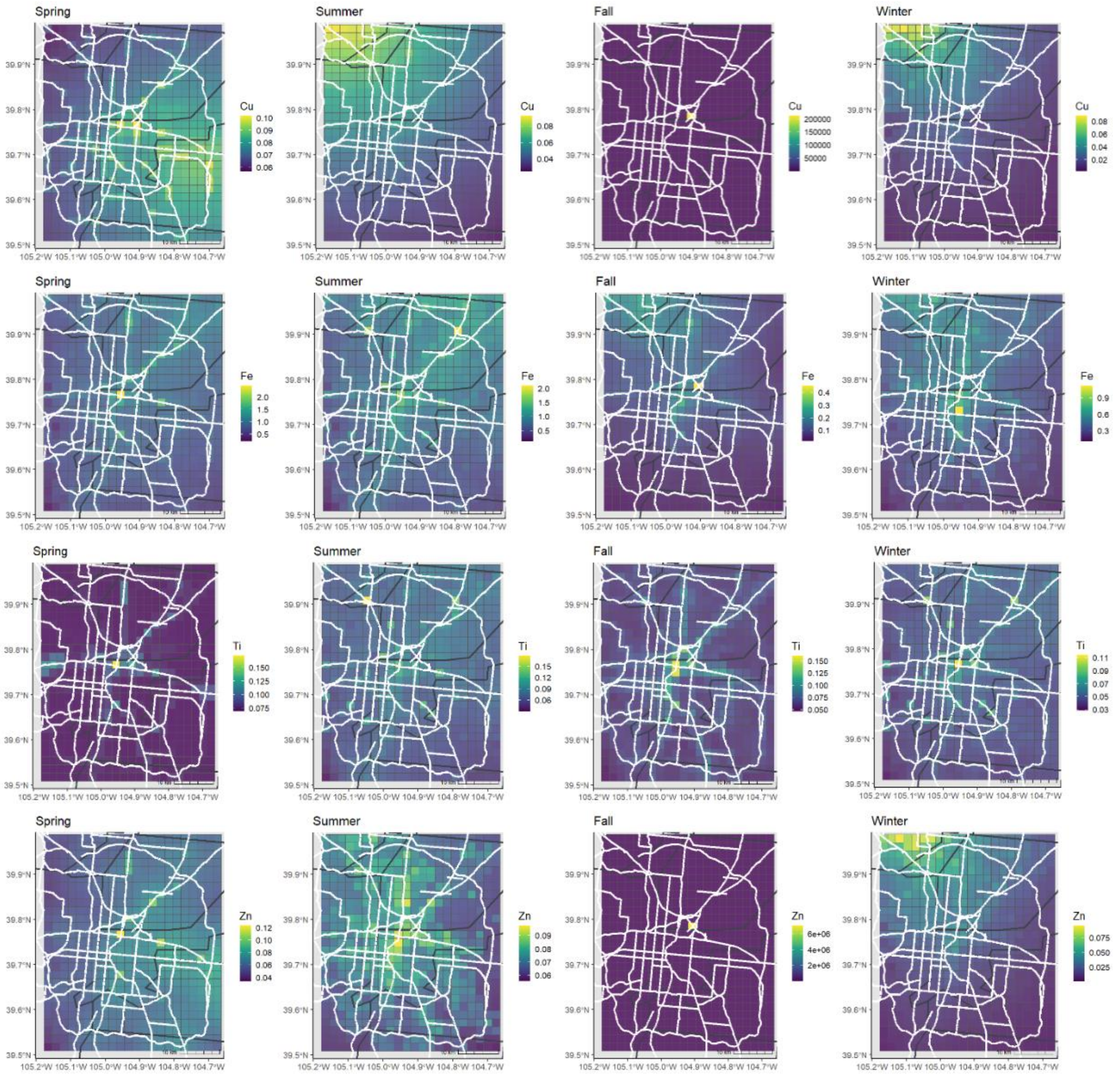

Figure S5. Estimated concentrations of Cu, Fe, Ti, and Zn by meteorological season, reported in units of ng/m^3, shown with county lines (black) and highways (white).
